# UV Resistance and Wetting of PLA Webs Obtained by Solution Blow Spinning

**DOI:** 10.3390/polym16172428

**Published:** 2024-08-27

**Authors:** Denys Baklan, Anna Bilousova, Miroslaw Wesolowski

**Affiliations:** 1 Department of Chemical Technology of Composite Materials, Chemical Technology Faculty, Igor Sikorsky Kyiv Polytechnic Institute, Beresteiskyi Ave. 37, 03056 Kyiv, Ukraine; a.bilousova@kpi.ua; 2 Department of Structural Mechanics, Faculty of Civil Engineering, Environmental and Geodetic Sciences, Koszalin University of Technology, ul. Sniadeckich 2, 75-453 Koszalin, Poland; miroslaw.wesolowski@tu.koszalin.pl

**Keywords:** solution blow spinning, nanofibers, PLA, UV stability, liquid repellency, Owens-Wendt approach

## Abstract

In this work, the resistance of polylactide-based non-wovens produced by solution blow spinning to environmental factors was investigated. An average contact angle of up to 136° was achieved with an average fiber diameter of 340 nm at the optimal material density and nozzle–substrate distance. When exposed to ultraviolet (UV) radiation, the polylactide non-wovens rapidly lose their hydrophobic properties due to changes in surface morphology resulting from fiber melting. It was demonstrated that the influence of surface structural features on hydrophobicity is greater than that of the material itself. The stability of the wetting properties under UV irradiation was assessed using the derivative parameters of the Owens–Wendt technique, which can serve as an additional method for estimating surface polarity.

## 1. Introduction

Massive plastic pollution is devastating ecosystems and degrading the quality of life, making it a critical environmental problem. According to the latest data, the World Health Organization (WHO) estimates that approximately 430 million tons of various plastics are produced annually. If production continues at the current rate, this figure is projected to triple by 2060 [1,2]. In the search for sustainable alternatives, new materials are being explored to replace conventional plastics. Polylactide (PLA) has emerged as a promising candidate due to its biodegradability, biocompatibility [3], physical, and mechanical properties that are comparable to those of traditional plastics. PLA remains stable at low temperatures, exhibits moderate oxygen and water permeability, and demonstrates significant resistance to fats and oils [4]. Additionally, PLA is easily recyclable, suitable for 3D printing, and can be blended with petroleum-based polymers, broadening its application in the food packaging, automotive, and medical fields [5]. However, its limited temperature resistance [6] and low ultraviolet (UV) resistance [7] constrain the broader use of PLA, making it imperative to investigate and enhance its performance for applications in other sectors.

A promising area for expanding the applications of PLA is in fiber-based materials with unique properties, among which nanofibers hold a significant position [8,9]. Nanofibers are one-dimensional structures with diameters less than 100 nanometers [10]. Biodegradable nanofibers represent an emerging class of materials with a wide range of applications in biomedical sensors, tissue engineering, and regenerative medicine [11,12,13].

Several techniques exist for the production of nanofibers, including melt spinning, jet spinning, wet spinning, and solution blow spinning (SBS) [14]. Among these, electrospinning is a well-known method [15,16,17]; however, it has limitations such as low productivity, challenges in scaling, and the requirement for high voltage [18,19]. A simpler alternative to electrospinning is solution blow spinning (SBS), in which fibers are formed from a polymer solution that is dried in a stream of pressurized gas or air. The air stream is directed through a nozzle parallel to the solution, enabling increased fiber production rates by adjusting the air volume [20]. The advantages of this method include the simplicity of the equipment and low production cost, making it a promising technology for micro- and nanofiber production. However, the SBS method has the drawback that the solvent evaporates during the fiber formation process, which can have a negative environmental impact [21]. This issue can be mitigated by replacing conventional solvents with more environmentally friendly and bio-based solvents, such as 2-methyltetrahydrofuran or dimethyl carbonate [22,23].

The properties of fibers can be controlled by adjusting air pressure, air volume, polymer concentration, and nozzle diameter. Optimal results are achieved using low-boiling-point solvents to ensure that the fibers are fully dry before reaching the substrate. Additional options include increasing the temperature of the air stream, ambient temperature, and the distance between the collector (substrate) and nozzle [24]. High air pressure improves fiber uniformity and reduces fiber diameter due to accelerated solvent evaporation while low pressure can cause turbulence and fiber twisting [25,26]. The air pressure must be maintained at the optimal level to ensure stable flow but below the threshold at which fiber agglomeration occurs due to insufficient solvent evaporation [27].

Materials based on PLA micro- and nanofibers exhibit hydrophobic properties with water contact angles of 142° [28] and 136° [29]. However, the surface free energy and its components have not been analyzed so far. Furthermore, previous studies have not examined the impact of PLA fiber production parameters (e.g., distance from the nozzle to substrate, pressure, solution concentration) on the contact angle of liquids. A detailed investigation of these characteristics could optimize the solution blow spinning process to produce materials with superhydrophobic properties, achieving a contact angle of more than 150°. Such fibrous materials can be used for effective oil/water separation [30] or dye adsorption [31].

Studies on the UV irradiation resistance of PLA have been extensively documented [32,33,34]. UV exposure accelerates the degradation of PLA-based films, a process that increases at high temperatures and relative humidity [35]. However, there is a lack of data on the UV irradiation resistance of PLA fibers produced via the SBS method. Given the high contact angles of PLA fibers, it is also crucial to investigate their wettability and how this property changes when exposed to UV radiation. These studies are particularly important for understanding the durability and stability of PLA non-wovens.

The aim of this work is to examine the effect of UV irradiation on the hydrophobicity of polylactide (PLA) nanofibers obtained by the SBS method. The solution blow spinning parameters were selected based on previous studies [36,37] to produce thin and statistically uniform fibers. A modified Owens–Wendt approach, as previously described in [38], was used to quantitatively analyze the changes in wettability after UV irradiation. This study focuses on the alterations in the surface of the fibers under UV exposure and compares these changes in exposure with those induced by water, temperature, and the combined effects of UV and water to evaluate their durability and stability under various operating conditions.

## 2. Materials and Methods

### 2.1. Materials

Polylactic acid (PLA) Resin 4032D with Mw = 100,000–120,000, a density of 1.24 g/cm^3^, MFR (210 °C, 2.16 kg) 7 ± 0.5 g/10 min (Natureworks, Minnetonka, MN, USA), and glass transition temperature of 55–60 °C were used in this work. Dichloromethane (Merck, Darmstadt, Germany) was used as the solvent.

### 2.2. Preparation of PLA Solution Blow Spinning Non-Wovens

The non-woven materials were prepared following this procedure: First, a 5 wt.% PLA solution in dichloromethane was prepared by adding PLA pellets to the solvent and mixing on a magnetic stirrer for 4 h in a closed container. Next, 5 mL of the solution was poured into the reservoir of an airbrush (model 80-898, MIOL, Kyiv, Ukraine) with a 300 μm nozzle diameter. The solution was sprayed at room temperature onto a glass surface (76 mm × 25 mm). The collector was positioned 150 mm from the nozzle. Solution blow spinning was performed at a constant pressure of 4 bar, and the spraying time varied depending on the desired density of the material. After spraying, the material was dried and stored in a dry desiccator at room temperature. The density of the samples was determined as the ratio of the material mass on the glass substrate to the substrate area (g/m^2^).

### 2.3. Testing Procedures

#### 2.3.1. UV Exposure Test

UV resistance testing was performed in accordance with ASTM D4329 (https://www.astm.org/d4329-21.html, (accessed on 1 July 2024)) and related procedures ASTM G154 (https://www.astm.org/g0154-23.html, (accessed on 1 July 2024)) and G151 (https://www.astm.org/g0151-19.html (accessed on 1 July 2024)). A 250 W UV lamp with an irradiance of 0.7 W/m^2^ (UVA 340) was used for the test. The procedure was modified to eliminate the effects of moisture and to evaluate UV resistance only. Material samples were placed on a steel substrate 50 cm from the UV lamp to improve heat dissipation. The samples were allowed to cool to room temperature for 2 min before contact angles were measured. All UV exposure tests were performed on the same day to minimize the influence of external factors.

#### 2.3.2. Immersion in Water

To assess water resistance, material samples were submerged in a container of distilled water at room temperature. After the designated exposure time, the samples were removed and dried at 35–40 °C for 10 min.

#### 2.3.3. Combined Water Immersion and UV Exposure Test

For the combined test, a container of distilled water was placed in the UV chamber described in Section 2.3.2, with the water level maintained at 1 mm above the samples. At the end of the test, the samples were removed and dried at 35–40 °C for 10 min.

#### 2.3.4. Temperature Resistance

To evaluate the effect of temperature, material samples were placed in a forced-air convection drying cabinet. At each selected temperature, the samples were held for 1 h. After the test, the samples were removed and cooled to room temperature.

### 2.4. Characterization

#### 2.4.1. Infrared Spectroscopy

IR spectra were recorded on a Nicolet 4700 FTIR spectrometer (Thermo Fisher Scientific, Waltham, MA, USA) in the 4000–400 cm^−1^ range in transmission mode (16 scans, 1 cm^−1^ resolution). To obtain the spectra of SBS PLA material, the material was deposited on rings with a diameter of 20 mm. The carbonyl index was calculated as the ratio of the C=O peak intensity (1750–1760 cm^−1^) to the C–H reference peak at 1450–1460 cm^−1^ [39].

#### 2.4.2. Surface Characterization

The surface topography of the obtained non-woven materials was studied using a MIRA3 LMU scanning electron microscope (Tescan, Brno, Czech Republic). All images were captured at an accelerating voltage of 10 kV and a current of 12 pA. To reduce surface charging, the samples were coated with a 15 nm layer of gold using a precision coating and etching system (682 PECS, Gatan, Inc., Pleasanton, CA, USA). The contact angles of the materials were determined using a digital camera and optical microscope (Delta Optical HCDE-50 digital camera, Delta Optical, Shanghai, China) and ScopeTek View software (version 1.0.0.1, ScopeTek Optics Electronics, Hangzhou, China) according to the method described in [40]. A micropipette was used to accurately dispense the volume of a drop of test fluid. The 5 µL droplets were applied at five different points on the sample surface. After measuring the contact angles, the samples were dried at 30–35 °C for 5 min.

#### 2.4.3. Surface Energy and Owens–Wendt Characterization

Water and water–ethanol mixtures were used as test liquids to increase the measurement resolution. The surface tension of the test liquids was calculated using the method described in [41].

In study [38], it was shown that the classic Owens–Wendt approach is not applicable to rough surfaces, including fibrous surfaces. Distortions of the linear shape of the Owens–Wendt diagram for such surfaces can be used as a tool to characterize the Cassie state and to evaluate the stability of surface wetting. Derivative parameters [42] have been used to numerically describe the wettability of textured surfaces. The parameter $\sigma_{TS}^{D}/\sigma_{S}^{D}$ expresses the quality of water repellency of the textured surface and indicates the minimum proportion of dispersion interaction of the material surface compared to the flat surface. The parameter $\sigma_{LIN}^{P}$ shows the value of the polar component of the test liquid at which linearization of the Owens–Wendt plot occurs. $\sigma_{LIN}^{P}$ is sensitive to the polar component of the surface free energy, which indicates the repellency of polar fluids.

## 3. Results

**Figure 1:**
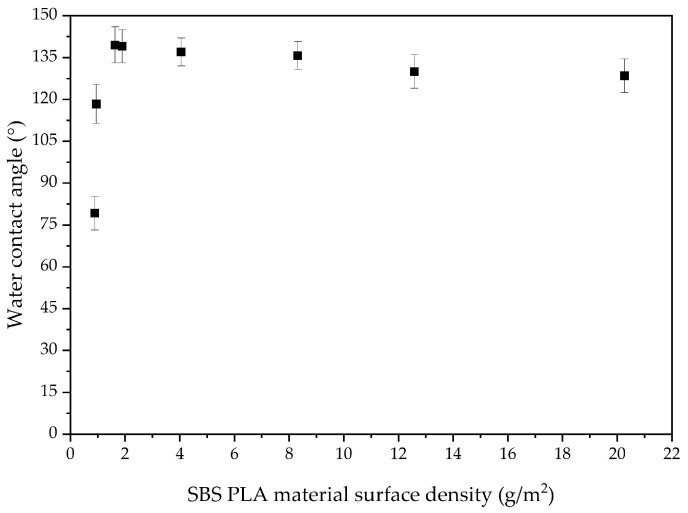
Effect of SBS-PLA material density on water contact angle.

The obtained materials demonstrate a correlation between contact angles and density (Figure 1), which is determined by sputtering time. As shown in Figure 1, the highest water contact angle values are observed at a density of 1.5–2 g/m^2^. A gradual decrease in the contact angle follows this peak, which can be attributed to an increased proportion of water droplets in contact with the polymer fibers. At densities below 1.5 g/m^2^, the water droplets also contact the glass surface, leading to a slight decrease in the contact angle.

The optimal distance from the nozzle to the glass substrate was determined experimentally using a 5 wt.% PLA solution in dichloromethane and a nozzle diameter of 0.3 mm (Table 1). The highest water contact angle values were observed at a distance of 15 cm and averaged 136°. Consequently, all materials in this study were obtained at this distance.

For the study of PLA-based non-wovens, different densities of 1, 2, 5, and 10 ± 0.1 g/m^2^ were selected, with the samples labeled as P1, P2, P5, and P10, respectively. The PLA film sample was used as a reference for comparison and labeled as “PLA film”.

### 3.1. Effect of Various Environmental Factors on the Contact Angle of SBS PLA Materials

**Figure 2:**
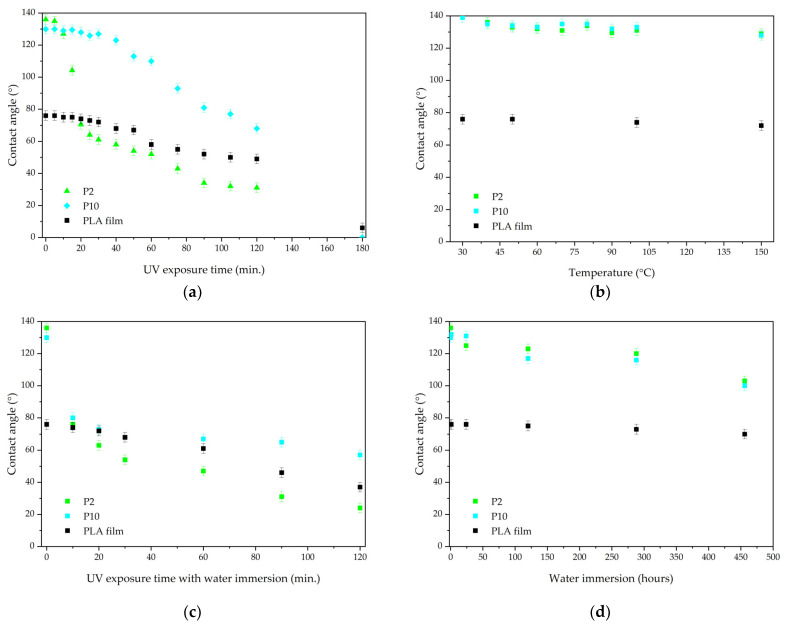
Water contact angles after (**a**) UV radiation; (**b**) temperature; (**c**) water and UV radiation; and (**d**) water.

Figure 2 shows that sample P2 has a contact angle of 136°, P10 has a contact angle of 130°, and the PLA film has a contact angle of 76°. The contact angle decreases for all materials after UV exposure (Figure 2a). The 2 g/m^2^ material loses hydrophobicity after 15 min of UV exposure while the 10 g/m^2^ sample is more resistant, losing hydrophobicity only after 75 min. The PLA film exhibits a gradual decrease in contact angle under UV irradiation. After 120 min, the contact angles were 31° for sample P2, 68° for P10, and 49° for the PLA film. Complete hydrophilization occurs after 180 min of UV exposure.

The effect of temperature on the contact angle is negligible (Figure 2b). After 1 h of exposure at 150 °C, a slight decrease in contact angle is observed for samples P2 and P10, which have angles of 129° and 128°, respectively. At the same time, the PLA film’s contact angle changes to 72°.

Exposure to water also has a minimal effect on the contact angle (Figure 2d). Even after more than 450 h of water immersion, the materials retain their hydrophobic properties: the water contact angles are 103° and 100° for samples P2 and P10, respectively. Meanwhile, the PLA film exhibits a contact angle of 70°.

Exposure to water combined with UV radiation results in a greater decrease in contact angle than exposure to these factors separately (Figure 2c). The loss of hydrophobicity occurs after 10 min of testing. The 10 g/m^2^ sample is more resistant: after 120 min of exposure, the contact angle is 57°, while sample P2 has an angle of 24°. The PLA film has a contact angle of 37°.

### 3.2. FTIR Characterization

**Figure 3:**
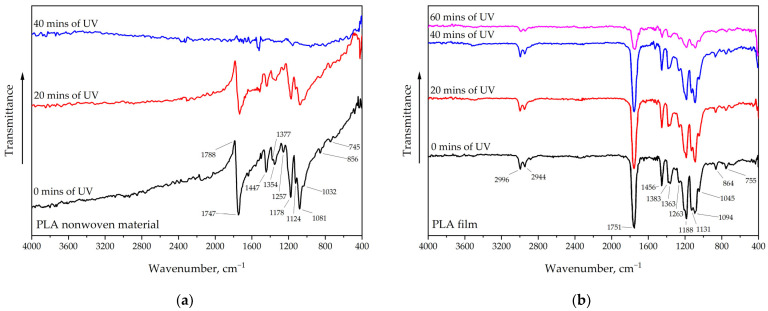
FTIR spectra after UV exposure: (**a**) SBS PLA material and (**b**) PLA film.

The FTIR spectrum of PLA (Figure 3) shows several characteristic absorption bands that allow the identification of the polymer’s main functional groups. The absorption bands at 2996 cm^−1^ and 2941 cm^−1^ correspond to asymmetric and symmetric C–H bond stretches of methyl groups (–CH_3_) characteristic of PLA [43].

The high-intensity absorption band at 1751 cm^−1^ reflects the stretching vibrations of the carbonyl group (C=O) of esters in the polymer. The band at 864 cm^−1^ indicates stretching vibrations of the C–C bond, which is also characteristic of PLA. The band at 1456 cm^−1^ represents stretching vibrations of the C–H bonds of methyl groups (CH_3_) while the peaks at 1383–1359 cm^−1^ are due to symmetric bending vibrations of C–H. The bands at 1188 cm^−1^ and 1094 cm^−1^ correspond to symmetric C–O–C stretching vibrations in complex ester bonds [44] while the peak at 755 cm^−1^ corresponds to C=O vibrations [45]. The carbonyl indices of the PLA film are shown in Table 2. The calculated carbonyl indices show that under UV exposure, there is a gradual increase in the polarity of the PLA surface due to oxidation. The decrease in carbonyl index value after 60 min of UV exposure is most likely due to partial material degradation and the denudation of areas not yet exposed to UV.

The spectra of PLA fibers and films show identical peaks characteristic of this polymer. The main difference is the absence of the band at 2998–2944 cm^−1^ in the spectrum of PLA fibers. Additionally, a peak at 1788 cm^−1^ with negative intensity is observed in this spectrum, which may be related to PLA crystallization [46,47].

The degradation of PLA, both in the fibers and in the film, is not accompanied by the appearance of new peaks but only by a decrease in their intensity [48]. It has been observed that PLA-based fibers degrade much faster than conventional film. To better characterize the change in chemical properties, the estimation of wetting parameters is more appropriate, as changes in contact angles appear much earlier than changes in FTIR spectra [49].

The decrease in intensity of the entire IR spectrum is related to PLA degradation and fragmentation, which occurs at 40 min for fibers and at 60 min of UV exposure for film. The observed increase in peak intensity at 1094 cm^−1^ indicates changes in the PLA ether bond during aging, which is supported by the results from [50].

### 3.3. Morphological Changes in Materials

**Figure 4:**
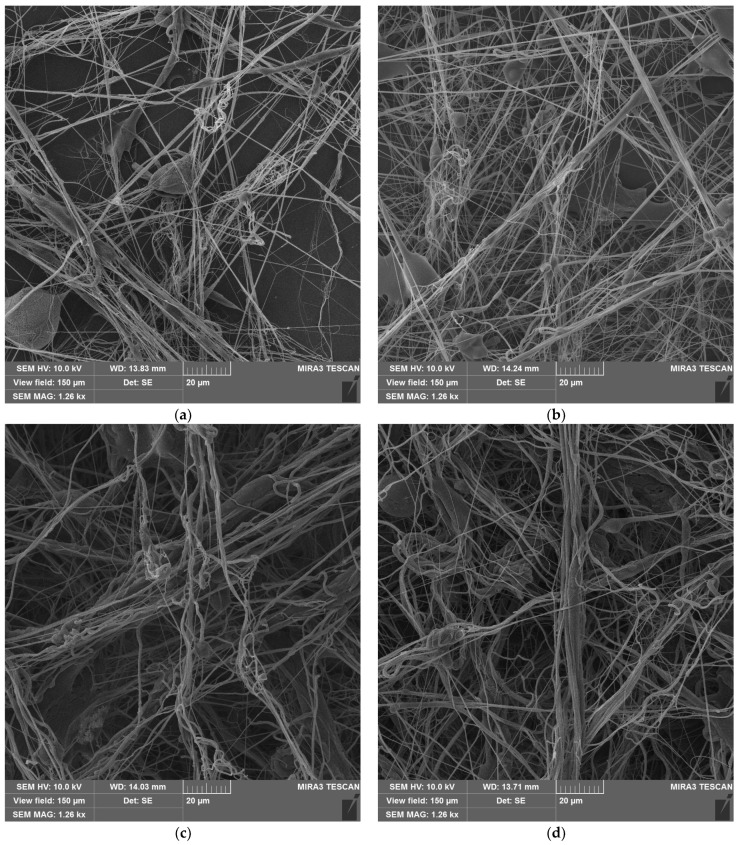
SBS PLA fiber materials with different densities before UV exposure: (**a**) sample P1; (**b**) sample P2; (**c**) sample P5; (**d**) sample P10.

SEM images were taken for samples with different densities (P1–P10) (Figure 4). The images show that all samples have a uniform structure consisting of thin fibers. All samples are composed of different types of fibers: single thin fibers, strands of several glued fibers, and thick fibers.

**Figure 5:**
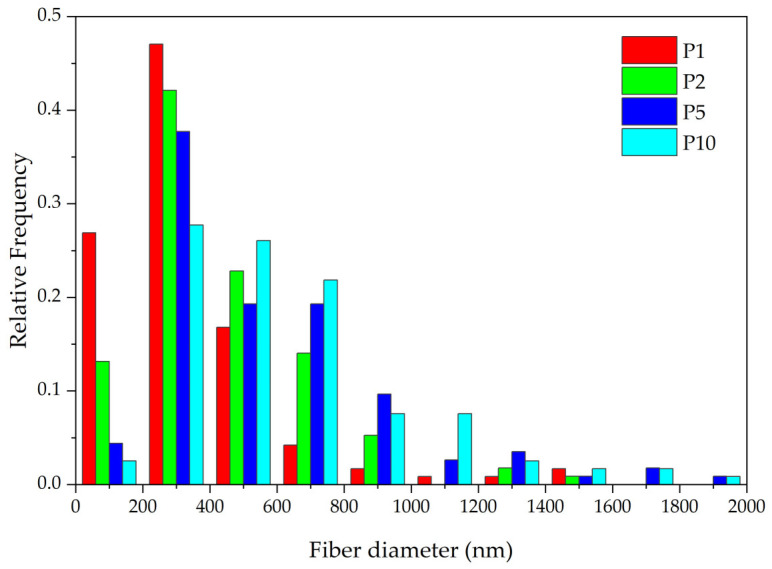
SBS PLA material fiber diameter distribution.

The average fiber size depends on the SBS PLA material density: 340 nm for P1, 452 nm for P2, 630 nm for P5, and 760 nm for P10. The size distribution curves are shown in Figure 5. The fiber size distribution also becomes broader with increasing density. However, thin fibers with diameters down to 40 nm are also present. The images also reveal bubbles on the fibers (Figure 4), which formed when the PLA solution was blown out. Bubbles are most common in low-density samples (P1, P2).

At higher densities, the fibers are more often bonded in the longitudinal direction, with the thickness of these bundles reaching 15 μm. It is also characteristic that with increasing density, more twisted fibers are observed, and their diameters increase.

**Figure 6:**
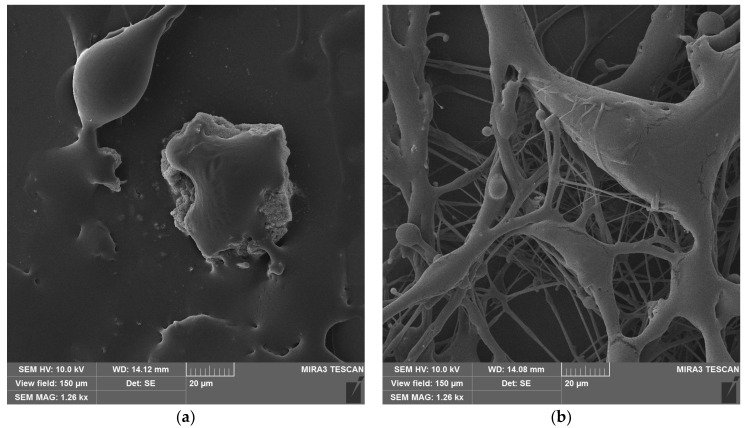
SBS PLA fiber materials with different densities after UV exposure: (**a**) sample P2 and (**b**) sample P10.

The SEM images of the materials after exposure to UV radiation (Figure 6) show that all PLA fibers have melted, and the microstructure of sample P2 is destroyed, explaining the contact angle decrease. Sample P10, however, has a partially preserved microstructure. The fibers deeper within the material remained intact or were only partially degraded, but fibers on the surface have melted or fused together.

### 3.4. Owens-Wendt Characterization

**Figure 7:**
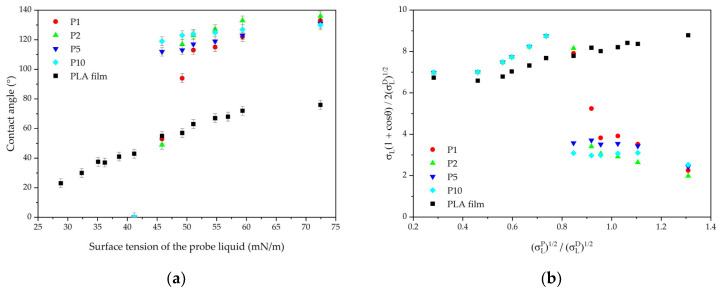
Wetting parameters of SBS PLA fiber materials: (**a**) contact angles and (**b**) Owens–Wendt plots.

The Owens–Wendt curves (Figure 7) for the fibrous materials have a non-linear shape, making it impossible to accurately determine the surface free energy and its components. For the PLA film, it was determined that the surface energy disperse component is 32.8 mN/m and the polar component is 5.8 mN/m, which aligns with values reported in the literature [51].

The sample with a density of 1 g/m^2^ has the lowest resistance to liquids with low surface tension compared to the other materials (Figure 7a). A sharp drop in resistance is observed when exposed to liquids with a surface tension below 50 mN/m. Increasing the material density enhances the Cassie state stability, improving resistance up to 41 mN/m. Materials with a density greater than 5 g/m^2^ demonstrate better stability, although the water contact angle is lower than for sample P2.

**Figure 8:**
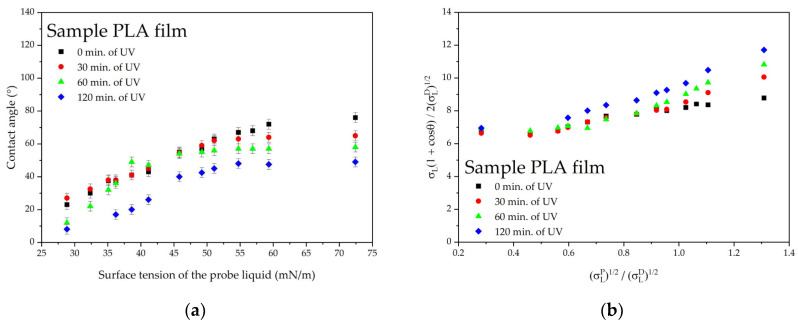
Wetting parameters of PLA film after UV irradiation: (**a**) contact angles and (**b**) Owens–Wendt plots.

The surface energy of the PLA film increases under UV exposure, and a slight decrease in the contact angle is observed (Figure 8). The polar component contributes the most to this increase, while the dispersive component decreases insignificantly (Table 3).

**Figure 9:**
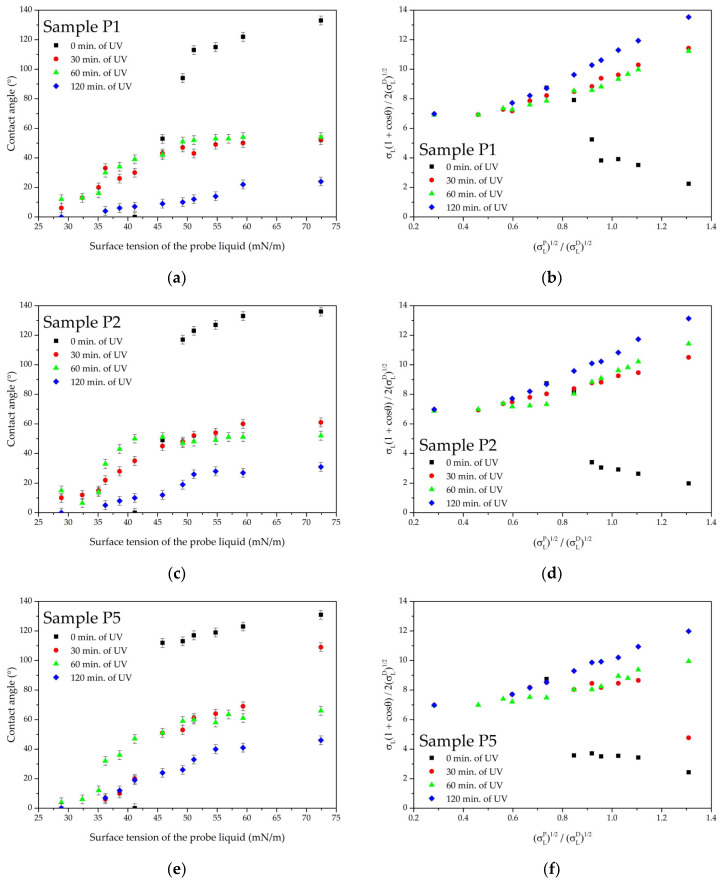

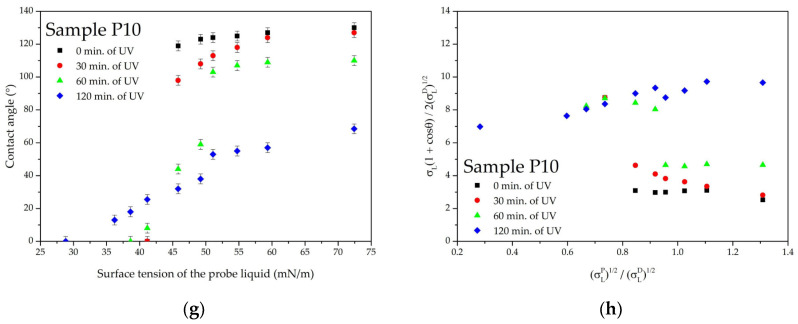
Wetting parameters of SBS PLA fiber materials with different densities after UV irradiation: (**a**) contact angles for sample P1; (**b**) Owens–Wendt plot for sample P1; (**c**) contact angles for sample P2; (**d**) Owens–Wendt plot for sample P2; (**e**) contact angles for sample P5; (**f**) Owens–Wendt plot for sample P5; (**g**) contact angles for sample P10; (**h**) Owens–Wendt plot for sample P10.

PLA-based non-wovens quickly lose their hydrophobic properties after UV irradiation (Figure 9). However, increasing material density can enhance UV stability. Samples with densities of 1–2 g/m^2^ lose hydrophobicity after 30 min of UV exposure, the sample with a density of 5 g/m^2^ after 60 min, and the sample with a density of 10 g/m^2^ after 120 min.

The analysis of the derivative parameter $\sigma_{TS}^{D}/\sigma_{S}^{D}$ for non-woven materials shows that the loss of hydrophobicity occurs in stages (Table 4). The first stage occurs at values of $\sigma_{TS}^{D}/\sigma_{S}^{D}$ up to 1 while the water contact angle remains above 90°. The second stage is the transition from the Cassie state to the Wenzel state, which is accompanied by a gradual linearization of the Owens–Wendt curve. This second stage occurs at values of $\sigma_{TS}^{D}/\sigma_{S}^{D}$ above 1.

The analysis of the derivative parameter $\sigma_{LIN}^{P}$ shows the test fluid polar component value below the Owens–Wendt plot becomes linear (Table 5). The value of $\sigma_{LIN}^{P}$ increases with UV exposure until the Owens–Wendt plot becomes completely linear. For denser samples, the parameter $\sigma_{LIN}^{P}$ remains at low values longer under UV exposure, indicating lower surface polarity.

## 4. Discussion

PLA-based non-wovens show low contact angle resistance with liquids when exposed to UV radiation. The primary reason for the loss of hydrophobicity is the surface topography change caused by fiber melting during UV exposure. As a result, the non-woven material loses its structural features and becomes more similar to the PLA film, as confirmed by the linearization in the Owens–Wendt plot and SEM images.

UV stability tests were conducted on a glass substrate at a temperature of 40 °C. Additional tests did not show a significant effect of high temperature on the contact angle or fiber structure. However, the infrared radiation from the lamp does affect the PLA fibers, causing them to heat up. Given the low thermal conductivity of PLA [52], the fibers can heat to temperatures higher than the substrate temperature, leading to their melting and settling on the substrate. A similar effect was reported in [53], where the gelation of fibers was observed under UV radiation. Additionally, UV degradation causes structural changes in the polymer, particularly the PLA ester bond transformation. Studies [45,47,48] have shown that the melting point and glass transition temperature of PLA decrease after UV aging. Even short irradiation times (20–60 min) are sufficient to influence these parameters, as confirmed by data from [47].

Derivative parameter $\sigma_{TS}^{D}/\sigma_{S}^{D}$ from the Owens–Wendt plot indicates that for low-density SBS PLA materials (up to 2 g/m^2^), the loss of structural features occurs within the first 30 min of UV exposure. After this point, it is no longer possible to calculate the $\sigma_{LIN}^{P}$ parameter for such surfaces. Increasing the material density enhances the hydrophobic properties resistance, with only a minor reduction in the water contact angle maximum values.

For the long-term preservation of hydrophobic properties, high-density materials are preferable, though they exhibit a slightly reduced water contact angle compared to low-density materials. To improve UV resistance, the use of nanoscale additives, such as UV-absorbing quantum dots, inorganic nanoparticles, or organic UV absorbers [34], which can be bio-based [54], could be considered. However, the nanoparticle use may negatively impact contact angle values. This was demonstrated in [55], where polymer polar degradation products remained on the nanoparticles. Another approach could involve further increasing the contact angle to superhydrophobic levels, creating a buffer to maintain the material’s water-repellent properties.

Applying this mechanism could lead to improvements in PLA fiber compositions and production methods, enhancing the hydrophobic properties’ stability under environmental conditions.

## 5. Conclusions

In this paper, the resistance of PLA fibers obtained by solution blow spinning was analyzed. It was found that at an optimal material density, a water contact angle of 136° can be achieved. However, these materials are not resistant to UV radiation and quickly lose their hydrophobic properties. The primary cause of this morphological change is the melting of thin PLA fibers, which leads to a gradual increase in the contact area between water droplets and the polymer surface. Additionally, a slight increase in the polymer’s polarity contributes to this effect. The density of the SBS PLA material significantly influences UV resistance, though there is a slight decrease in the water contact angle as the density increases. The use of the derivative parameters $\sigma_{TS}^{D}/\sigma_{S}^{D}$ and $\sigma_{LIN}^{P}$ from the Owens–Wendt plot allows a numerical comparison of the stability of the Cassie state and the water repellency of fiber-based surfaces. Overall, the change in morphology due to UV degradation has a more substantial impact on the contact angle of liquids than changes in polymer polarity.

## Figures and Tables

**Table 1:** Effect of nozzle–substrate distance on contact angle.

Parameter	Value			
Distance from nozzle to substrate, cm	5	10	15	20
Contact angle, °	132 ± 3	133 ± 3	136 ± 3	135 ± 3

**Table 2:** Carbonyl index values of PLA films.

UV Exposure Time, min	Values of Carbonyl Index	
	SBS PLA Material	PLA Film
0	2.58	2.56
20	2.64	2.62
40	-	2.71
60	-	2.64

**Table 3:** Surface energy of PLA film surface.

Sample	UV Exposure Time, min			
	0	30	60	120
σ	38.6	37.6	40.1	47.2
σ^D^	32.8	25.3	22.7	24.9
σ^P^	5.8	12.3	17.4	22.3

**Table 4:** The $\sigma_{TS}^{D}/\sigma_{S}^{D}$ parameters of non-woven materials.

Sample	UV Exposure Time, min			
	0	30	60	120
P1	0.15	5.17	5.56	7.34
P2	0.12	4.37	5.76	6.92
P5	0.18	0.90	4.37	5.76
P10	0.19	0.31	0.96	3.74

**Table 5:** The $\sigma_{LIN}^{P}$ parameters of non-woven materials.

Sample	UV Exposure Time, min			
	0	30	60	120
P1	19.2	-	-	-
P2	19.2	-	-	-
P5	14.4	33.1	-	-
P10	14.4	14.4	22.7	-

## Data Availability

The original contributions presented in the study are included in the article, further inquiries can be directed to the corresponding author.
